# Circulating Hsa-miR-431-5p as Potential Biomarker for Squamous Cell Vulvar Carcinoma and Its Premalignant Lesions

**DOI:** 10.3390/diagnostics11091706

**Published:** 2021-09-17

**Authors:** Mateusz Bujko, Kamil Zalewski, Martyna Szczyrek, Artur Kowalik, Joanna Boresowicz, Angelika Długosz, Krzysztof Goryca, Stanisław Góźdź, Magdalena Kowalewska

**Affiliations:** 1 Department of Molecular and Translational Oncology, Maria Sklodowska-Curie National Research Institute of Oncology, 02-781 Warsaw, Poland; Mateusz.Bujko@pib-nio.pl (M.B.); zalewski81@gmail.com (K.Z.); szczyrekmartyna@gmail.com (M.S.); joanna.boresowicz@gmail.com (J.B.); 2 Department of Gynecologic Oncology, Holycross Cancer Center, 25-734 Kielce, Poland; 3 Chair and Department of Obstetrics, Gynecology and Oncology, 2nd Faculty of Medicine, Medical University of Warsaw, 00-315 Warsaw, Poland; 4 Department of Molecular Diagnostics, Holycross Cancer Centre, 25-734 Kielce, Poland; Artur.Kowalik@onkol.kielce.pl; 5 Division of Medical Biology, Institute of Biology Jan Kochanowski University, 25-406 Kielce, Poland; 6 Department of Immunology, Biochemistry and Nutrition, Medical University of Warsaw, 02-091 Warsaw, Poland; adlugosz@wum.edu.pl; 7 Genomics Core Facility, Centre of New Technologies, University of Warsaw, 02-097 Warsaw, Poland; kgoryca@gmail.com; 8 Department of Clinical Oncology, Holycross Cancer Centre, 25-734 Kielce, Poland; stanislawgozdz1@gmail.com; 9 Collegium Medicum, Jan Kochanowski University, 25-317 Kielce, Poland

**Keywords:** vulvar carcinoma, high-grade squamous intraepithelial lesion, differentiated vulvar intraepithelial neoplasia, circulating microRNA, miR-431-5p

## Abstract

Vulvar squamous cell carcinoma (VSCC) develops from high-grade squamous intraepithelial lesions (HSIL) and differentiated vulvar intraepithelial neoplasia (dVIN). This study aimed to assess the diagnostic value of circulating hsa-miR-431-5p in vulvar precancers and VSCC. Expression levels of hsa-miR-431-5p were analyzed by quantitative RT-PCR in plasma samples of 29 patients with vulvar precancers (HSIL or dVIN), 107 with VSCC as well as 15 healthy blood donors. We used hsa-miR-93-5p and hsa-miR-425-5p as normalizers. The levels of miR-431-5p were increased in the blood of patients with VSCC compared to those with vulvar precancers. Statistically significant differences in the survival rates (time to progression) were revealed for VSCC patients categorized by miR-431-5p levels. Low levels of circulating miR-431-5p were found to be indicative of unfavorable survival rates. In summary, our data reveal the diagnostic potential of circulating miR-431-5p in patients with vulvar precancers and VSCC.

## 1. Introduction

Vulvar carcinoma is a genital malignancy with age-standardized incidence rates (ASR) ranging worldwide between 0.5 and 1.5 per 100,000. The current vulvar cancer ASR in Poland is 1.1, with 573 new cases diagnosed and 319 cancer-related deaths recorded in 2018 [1]. The American Cancer Society predicted 6120 cancers of the vulva to be diagnosed and more than 1550 patients to die due to vulvar cancer in the USA in 2021 [2]. According to SEER 18 data from 2011–2017, relative five-year survival rate for vulvar cancer patients is 71% for all disease stages combined [3].

Vulvar squamous cell carcinoma (VSCC) represents approximately 90% of vulvar cancer cases. VSCC develops on the basis of either the precancerous lesion: high-grade squamous intraepithelial lesion (HSIL), or the differentiated-type vulvar intraepithelial neoplasia (dVIN) [4]. The risk of progression from HSIL to VSCC is low and ranges from 3 to 16%, while for dVIN it reaches 32.8% [5]. In the United States, between 1973 and 2000, the incidence of vulvar precancers increased by 411% in contrast with increase of 20% for invasive cancers [6] reaching the reported incidence of precancers of 2.86 per 100,000 women in 2000 [7]. Of patients diagnosed with these disorders, 75% are below 50 years of age [7].

There is no specific screening method to detect VSCC or its precursor lesions. Patients with vulvar precancerous lesions are asymptomatic in approximately 40% of cases and lesions are noted incidentally during pelvic examination [8]. These conditions may coexist in the same anatomical region. Therefore, a careful histological examination of tissue biopsy is indispensable for a definitive diagnosis as 3.2–18.8% of biopsies reveal unsuspected stromal invasion leading to diagnosis of invasive carcinoma [8,9]. Despite the constantly increasing number of new cases of vulvar precancers in younger patients [10] and unsatisfactory treatment outcomes, no markers allowing precise and non-invasive diagnosis and/or differentiation of malignant and premalignant lesions of the vulva have been developed to date. It is therefore of a great need to develop an effective, minimally invasive molecular test that would allow to diagnose early-stage VSCC or assess the risk of cancer development in patients with vulvar precancers.

MicroRNAs (miRNAs) are short, small, non-coding RNAs that regulate diverse aspects of cell physiology, and some were shown to be directly involved in oncogenesis, as either oncogenes or tumor suppressors. A number of miRNAs also affects the carcinogenesis of gynecological cancers [11], yet their profiles in vulvar cancers remain underappreciated [12]. To date, the most comprehensive studies on miRNAs in VSCC were by de Melo Maia et al. [13] and by Yang and Wu [14]. These studies shed some light on the miRNA signatures in vulvar carcinoma by identifying 79 and 157 miRNAs that showed expression alterations in tumors compared to control tissues.

miRNAs are released into the extracellular space and thus can be found at low levels in body fluids, including blood [15]. The potential of measuring circulating miRNAs as biomarkers for early diagnostics prognostics and support of clinical decisions in cancer management has been proved in hundreds of studies [15]. miR-431 is involved in tumor development, and its downregulation was proposed as a possible novel progression marker for renal cancer, lung cancer, medulloblastoma, glioblastoma, and melanoma, even at the early stage of cancer [16,17,18,19]. To our knowledge, the presence of miR-431-5p has not been examined in VSCC patients, neither in tumors nor blood. Our study aimed to determine miR-431-5p microRNAs circulating levels in patients with precancers and squamous cell carcinoma of the vulva, as well as to examine whether they would be of prognostic value.

## 2. Materials and Methods

### 2.1. Patients

Clinical material was obtained from 28 patients treated for vulvar premalignant lesions (HSIL, *n* = 21 and dVIN, *n* = 8) and VSCC (*n* = 107) at the Maria Sklodowska-Curie National Research Institute of Oncology Warsaw, Poland and at the Holycross Cancer Center in Kielce, Poland between December 2004 and April 2019. Enrolled in the study were 91 patients with microscopically confirmed primary VSCC at early (56 FIGO stage I, 3 FIGO stage II) and advanced stages (29 FIGO stage III, 3 FIGO stage IV). For six primary VSCC tumors, the FIGO stage was not reported. Additionally, nine VSCC patients with local recurrent VSCC were recruited. Basic demographic data of the patients are provided in Table 1. The control group was recruited from among the staff of the Holycross Cancer Center (*n* = 15).

### 2.2. HPV Genotyping

The HPV status was determined as described previously [20] using the AmpliSens HPV HCR-genotype-titre-FRT test (InterLabService Ltd., Moscow, Russia) in precancer lesions and vulvar malignant tumors.

### 2.3. RNA Isolation from Plasma and RT-qPCR

Plasma samples were obtained from patients and healthy donors were banked at −70 °C prior to their analysis. Total RNA was isolated from 200 µL of plasma using miRNeasy Serum/Plasma Kit (Qiagen, Hilden, Germany) and miRNA was reverse transcribed using miRCURY LNA RT Kit (Qiagen) according to the manufacturer’s protocol.

Expression levels of miR-431-5p were analyzed by quantitative RT-PCR (RT-qPCR) using the miRCURY LNA miRNA Custom PCR Panels (Qiagen) according to the manufacturer’s protocol. Reactions were performed in duplicates in 7500 Fast Real-Time PCR System (Applied Biosystems, Waltham, MA, USA) in a final volume of 10 µL reaction mix, according to the panels’ manufacturer instructions (Qiagen). The collected data were analyzed using threshold-cycle (Ct) values for the miRNAs with the SDS 2.1 software (Applied Biosystems). Results were normalized with hsa-miR-93-5p and hsa-miR-425-5p, as previously identified stable reference microRNAs [21]. The relative amounts of each miRNA in the plasma of patients with vulvar cancers and precancers as well as of the healthy donors were calculated by the 2−ΔCtT method. PCR nondetects were used in calculation of relative gene expression level with the use of nondetects R package [22].

### 2.4. Statistical Analyzes

The nonparametric Kruskal–Wallis test with the post-hoc Dunn’s multiple comparison test was used to assess the significance of differences in microRNA expression between the multiple sample groups. The Mann–Whitney test was used to compare the differences in microRNA levels between two sample groups. Association between the miRNA level with time to progression of the disease in patients with primary VSCC was determined with log-rank test and Kaplan-Meyer estimator. Statistical analyses were performed and the results were visualized using GraphPad Prism 6.07 (GraphPad Software, La Jolla, CA, USA). Differences at *p* < 0.05 were considered significant.

## 3. Results

**Figure 1:**
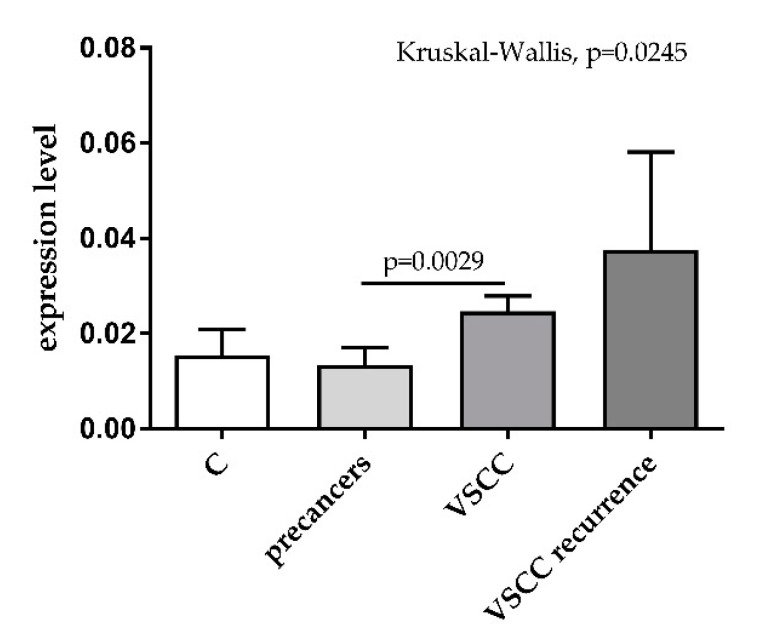
Expression levels of miR-431-5p in plasma samples of healthy blood donors (C, *n* = 15), patients with precancer lesions (*n* = 29), and patients with primary (VSCC, *n* = 98) and recurrent (VSCC recurrence, *n* = 9) vulvar malignant tumors.

Expression levels of miR-431-5p were analyzed by RT-qPCR in the plasma samples obtained from 29 patients with precancer lesions and 98 patients with VSCC. The levels differed significantly by the analyzed sample groups (*p* = 0.0245, Kruskal–Wallis test). The levels of miR-431-5p were increased in the blood of patients with VSCC as compared to those with vulvar precancers (Figure 1).

**Figure 2:**
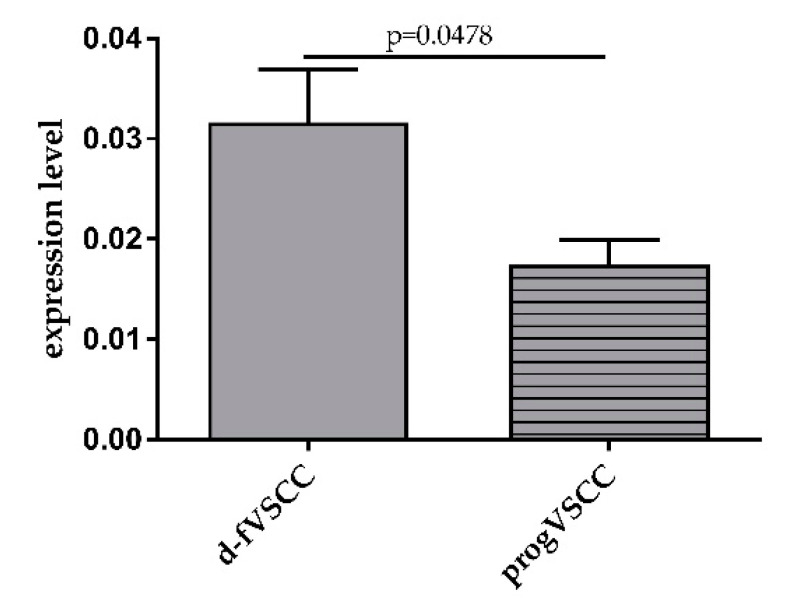
Comparison of normalized miR-431-5p expression levels in plasma obtained from patients with primary VSCC who progressed (progVSCC) and from those who were disease-free (d-fVSCC) during follow-up.

We then compared the results of the normalized expression levels of miR-431-5p obtained for patients with primary VSCC who progressed (progVSCC; 15.5 months of median follow-up time, 0–101 range) and from those who remained disease-free (d-fVSCC; 48.5 months of median follow-up time, 1–169 range) during follow-up. The data distinguished these two groups of VSCC patients (*p* = 0.0478) with circulating miR-431-5p levels being lower in the progVSCC sample group (Figure 2). No relationship between miR-431-5p levels and FIGO stage (FIGO I, *n* = 56; FIGO II, *n* = 3; FIGO III, *n* = 30; FIGO IV, *n* = 3; undetermined, *n* = 6), G score (G1, *n* = 32; G2, *n* = 37; G3, *n* = 13; undetermined, *n* = 16), nor hrHPV status (negative, *n* = 26; positive, *n* = 44; undetermined, *n* = 28) was found (data not shown).

**Figure 3:**
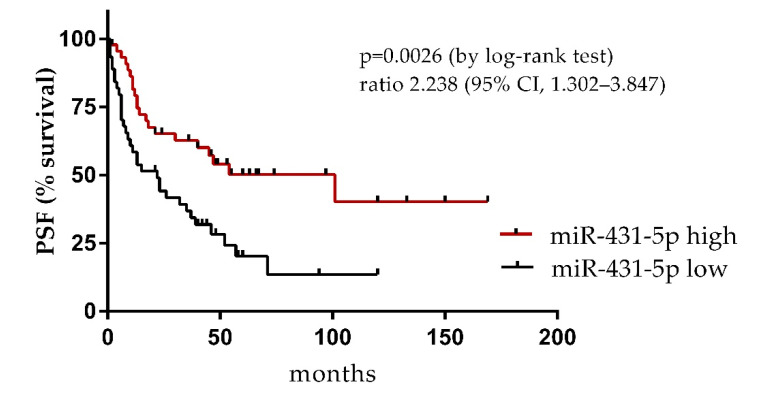
Survival curve (time to progression) according to the circulating miR-431-5p levels in patients with primary VSCC. Abbreviations: PFS, progression-free survival; high and low, high and low miR levels (> and < median normalized level, respectively).

Next, we examined the association between the miRNA levels and time to progression of the disease in patients with primary VSCC for which the follow-up data were available (*n* = 92). The follow-up times of the enrolled patients were determined from the date of surgery to the date of death or the date that the last interview was registered. Survival curves of the two groups of patients with primary VSCC tumors categorized by the miR-431-5p levels (with median expression used as cutoff value) are shown in Figure 3. Patients with lower miR-431-5p plasma levels had significantly shorter time to progression than the “miR-431-5p high group” (22 vs. 101 months, *p* = 0.0026).

## 4. Discussion

The relationship between miRNA expression for common malignancies of the female reproductive system, such as cervical, ovarian, and endometrial cancers, is well-documented [11,23]. Research on miRNA in rarer gynecological tumors still remains in its infancy. Nevertheless, early studies on non-coding RNAs have already revealed the importance of these molecules in vulvar carcinogenesis. In their initial studies on VSCC, de Melo Maia et al. [13] showed that deregulated expression of several identified miRNAs correlated with clinicopathological features such as the presence of lymph node metastasis (downregulation of miR-223-5p and miR-19-b1-5p), vascular invasion (downregulation of miR-100-3p and miR-19-b1-5p), HPV infection (upregulation of miR-1274b and downregulation of miR-519b-3p), or advanced FIGO stage (miR-519b and miR-133a overexpression). More recently, the upregulation of miR-20a and miR-106a was demonstrated to correlate with deeper tumor invasion in VSCC, providing the rationale—and promising preliminary results of in vitro experiments—for targeting the members of the miR-17 family as a novel miRNA-based therapeutic option against VSCC [24]. Other authors reported that upregulation of miR-590-5p in VSCC tumors is associated with lymphatic metastases [14].

Accumulating data on circulating miRNAs’ point to their potential as biomarkers of diagnostic and prognostic importance in cancers of gynecological origin [25,26]. Despite of the documented involvement of miRNAs in VSCC carcinogenesis, to the best of our knowledge, the circulating miRNAs in patients with VSCC or vulvar precancers have not been examined to date. Our results revealed increase in circulating miR-431-5p levels in the blood of patients with VSCC compared to those with vulvar precancers. However, a decrease of this miRNA was observed to be associated with aggressive course of VSCC. Importantly, the relationship between lowered miR-431-5p levels and poor survival outcome—in terms of significantly shortened time to progression—was determined.

miR-431-5p is downregulated in several types of cancer [27], having the antitumor effect by downregulating RAF1 oncogene (Raf-1 proto-oncogene, serine/threonine kinase) [28], ATG3 enzyme, autophagy regulator (Autophagy Related 3) [29] and tumor-promoting ATF3 (Activating Transcription Factor 3) [30] as demonstrated in studies on lung, colon, and tongue cancers, respectively. The role of miR-431 in gynecological cancers remains unknown. However, we hypothesized that this molecule could belong to extracellular miRNAs present in the blood of VSCC patients. This suppressor was previously reported to be downregulated in the blood of patients with colorectal cancer [31], diffuse large B-cell lymphoma [32], and papillary thyroid carcinoma (especially those with lymph node metastases) [33].

Our study reveals that expression levels of circulating miR-431-5p may potentially serve as biomarkers for differential diagnosis of VSCC and vulvar precancer lesions. Nonetheless, our study has some important limitations. Firstly, the small sample size of patients treated for vulvar premalignant lesions and VSCC. This is especially decisive for patients with precancerous lesions, for, as our results suggest, increased levels of miR-431-5p might be associated with increased risk of progression to vulvar cancer (contrary to VSCC in which lowered miR-431-5p plasma levels indicate a higher risk of progression). To be verified, this hypothesis requires both larger precancerous lesion sample sizes and longer patients’ follow-ups. Future validation studies should also examine their association with precancers, adjacent *lichen sclerosus*, and inflammatory dermatoses at the anatomical site of origin of the VSCC. Secondly, the functionality of the studied miRNA in gynecologic cancers as well as its source in patients’ circulations remain to be established. As a first step for VSCC, multivariate analysis of miR-431-5p abundance both in tumors and circulation with the established clinico-pathological features needs to be assessed [34,35].

## 5. Conclusions

Despite the fact that different medical and surgical management modalities are applied to prevent development of invasive vulvar carcinoma, vulvar precancer lesions recur in one-third of women, and approximately 4–8% of patients develop invasive carcinoma [8]. Therefore, long-term surveillance of the entire lower genital tract is mandatory. Our preliminary results support the utility of miR-431-5p levels examination in the blood as an non-invasive approach that would allow monitoring of patients with vulvar cancers, possibly to identify those at high risk of progression.

## Figures and Tables

**Table 1:** Basic demographic data of enrolled patients ^1^.

	Patients Enrolled (n)	Median Age (Years) (Range)	FIGO	G	hrHPV Status
HSIL	21	57.1 (26.3–83.4)			
dVIN	8	50.4 (18.8–79.5)			
Primary VSCC	107	72.4 (37.3–94.2)	I *n* = 56 II *n* = 3 III *n* = 30 IV *n* = 3 N/A *n* = 6	G1 *n* = 32 G2 *n* = 37 G3 *n* = 13 N/A *n* = 16	negative *n* = 26 positive *n* = 44 N/A *n* = 28
Recurrent VSCC	9	74.8 (85.8–72.1)			

^1^ dVIN, differentiated-type vulvar intraepithelial neoplasia; FIGO, the International Federation of Gynecology and Obstetrics; G, tumor grade; hrHPV, high-risk human papilloma virus; HSIL, high-grade squamous intraepithelial lesions; N/A, data not available.

## Data Availability

Data are available upon reasonable request from the corresponding author.
